# Neurological Side Effects of TNF-α Inhibitors Revisited: A Review of Case Reports

**DOI:** 10.3390/medicina60091409

**Published:** 2024-08-28

**Authors:** Armand Gogulescu, Alexandru Blidisel, Codruta Soica, Alexandra Mioc, Adrian Voicu, Alina Jojic, Mirela Voicu, Christian Banciu

**Affiliations:** 1 Faculty of Medicine, Victor Babes University of Medicine and Pharmacy, 2 Eftimie Murgu, 300041 Timisoara, Romania; gogulescu.armand@umft.ro (A.G.); banciu.christian@umft.ro (C.B.); 2 Faculty of Pharmacy, Victor Babes University of Medicine and Pharmacy, 2 Eftimie Murgu, 300041 Timisoara, Romania; codrutasoica@umft.ro (C.S.); alexandra.mioc@umft.ro (A.M.); adrian.voicu@umft.ro (A.V.); alina.jojic@umft.ro (A.J.);

**Keywords:** TNF-α inhibitors, neurological side effects, neurological events, neurological damage, demyelination

## Abstract

Over the past two decades, the use of tumor necrosis factor alpha (TNF-α) inhibitors has significantly improved the treatment of patients with immune-mediated inflammatory diseases. Firstly, introduced for rheumatoid arthritis, these inhibitors are currently approved and used for a variety of conditions, including ankylosing spondylitis, Crohn’s disease, juvenile idiopathic arthritis, psoriasis, psoriatic arthritis, ulcerative colitis, and chronic uveitis. Despite their immense therapeutic efficacy, TNF-α inhibitors have been associated with neurological adverse effects that bring new clinical challenges. The present review collects data from multiple studies to evaluate the incidence and the relationship between TNF-α inhibitors and neurological side effects and to explore the potential underlying mechanisms of this association. Moreover, it highlights the importance of patient selection, particularly in the case of individuals with a history of demyelinating diseases, raises awareness for clinicians, and calls for ongoing research that will improve TNF-α targeting strategies and offer safer and more effective therapeutic options.

## 1. Introduction

Although the treatment of immune-mediated inflammatory diseases is constantly improving, tumor necrosis factor alpha (TNF-α) inhibitors still represent the main therapeutic option, able to significantly alter the physical course of various pathologies. TNF-α inhibitors were introduced in therapy two decades ago as treatment against rheumatoid arthritis, but since then new indications have occurred; currently, TNF-α inhibitors are approved for use in ankylosing spondylitis (AS) [1], Crohn’s disease (CD) [2], juvenile idiopathic arthritis (JIA) [3], psoriasis (PSO) [4], psoriatic arthritis (PsA) [5], rheumatoid arthritis (RA) [6], ulcerative colitis (UC) [7], chronic uveitis [8], and others. 

Inhibitory effects against TNF-α were revealed for several molecules both natural and synthetic; an excellent review published in 2022 assessed the main natural compounds that may act as TNF-α inhibitors [9]. The authors introduced several alkaloids, flavonoids, terpenes, and polyphenols as potential TNF-α inhibitors and discussed their interactions with aminoacidic residues, which appear to be essential for their biologic activity; such data may provide future scaffolds for anti-inflammatory drugs. Among synthetic molecules, thalidomide was revealed as active against TNF-α through various mechanisms [10]; its derivatives, lenalidomide [11] and pomalidomide [12] also show TNF-α inhibitor effects that may be exploited in severe pathologies, including malignancies. Other molecules such as xanthine derivatives [13] or bupropion [14] were also identified with TNF-α inhibitor activity. However, most TNF-α inhibitors are monoclonal antibodies, with four such compounds being currently marketed—infliximab, adalimumab, golimumab, and certolizumab (Table 1); in addition, the fifth compound currently approved is etanercept, a fusion protein produced by the recombinant DNA technique. Biological therapy with TNF-α inhibitors has become more affordable once biosimilars were introduced; currently, the five marketed TNF-α inhibitors are on the WHO’s list of essential medicines. Research is ongoing on finding biomarkers that would allow better monitoring of the biological treatment; in addition, new delivery systems with TNF-α inhibitors may be designed in the future with or without associated agents.

Generally, TNF-α inhibitors are considered to display safe pharmacological profiles, being assessed even during pregnancy, where they cross the placenta starting from the 22nd gestational week [15], as well as during the COVID-19 epidemic, where patients under treatment with TNF-α inhibitors exhibited lower rates of morbidity and mortality related to the viral infection compared to the general population [16]. The most common reported adverse effects consist of headaches, rash, anemia, cough, diarrhea, and abdominal discomfort [17]. However, severe adverse reactions have also occurred, such as infections, including tuberculosis, and malignancies [18]. An excellent review with meta-analysis was published in 2021 by Li et al., who emphasized severe side effects occurred during treatment with TNF-α inhibitors; briefly, the meta-analysis suggested an increased risk of infectious events and various malignancies [18]. Controversially, another study, although smaller, found only an increased risk of lymphoma in inflammatory bowel disease patients under TNF-α inhibitor treatment [19]. Systemic lupus erythematosus (SLE) was reported as induced by biologic therapy of psoriasis, particularly in patients with psoriatic arthritis or with comorbidities such as rheumatoid arthritis [20]. The authors reported that the induced SLE can be correlated with the depletion of cytokines IL-2 and IL-10 and an overall cytokine imbalance; however, they concluded that SLE induction is drug-specific and less class-specific, therefore recommending a detailed examination of the patient in order to establish the potential of drug-induced SLE and the specific drug that may trigger the disease. Additionally, prolonged use of TNF-α inhibitors may cause in some patients the development of anti-drug antibodies (i.e., adalimumab), thus promoting adverse effects and reducing drug efficacy; other studies revealed that induced autoantibodies, clinically manifest autoimmune diseases, and paradoxical inflammation may also occur as a result of administered TNF-α inhibitors [21].

In terms of neurological side effects, the literature reports a limited number of cases where such effects appeared, but some consisted of dramatic symptomatology or even the triggering of life-threatening pathologies. This review aims to summarize the most significant data on the neurological damage that may be caused by the administration of TNF-α inhibitors; it will also attempt to distinguish between cases where neurological reactions occurred as a result of the biological treatment and those where they can be regarded as a simultaneous separate condition.

**Table 1:** Anti-TNF-α agents.

	Type	Mechanism of Action	Date of Approval (FDA)	Approved Indications	Administration	Pharmacodynamics	Ref.
Etanercept	Dimeric soluble fusion protein—the extracellular ligand-binding portion of the human p75 TNFR (TNFR2) linked to the Fc portion of human IgG1	Inhibits the binding of TNF-α and TNF-β to cell surface TNFRs → renders TNF biologically inactive.	1998	RA, JIA, PsA, PsO, AS, nr-axSpA	Subcutaneously weekly	Modulates the biological response/decrease in serum levels of E-selectin, ICAM-1, VCAM-1, VEGF, IL-6, IL-1 Down-regulates the serum levels of MMP-1 and MMP-3	[22,23,24,25,26]
Infliximab	Chimeric IG1κ monoclonal antibody (75% human and 25% mouse)	Selectively neutralizes the biological activity of TNFα by binding to the tmTNF-α + sTNF-α → inhibits the binding of TNFα to TNFR. No binding/inactivation of TNF-β	1998	AS, CD, JIA, PsO, PsA, RA, UC, nr-axSpA	Intravenously every 6–8 weeks	Decreases serum levels of IL-6 and CRP Reduced the T-cell number in blood vessels + synovium + psoriatic skin lesions Reduced the macrophages in the synovium Modulates the biological response/decrease in serum levels of E-selectin, ICAM-1, VCAM-1, IL-8, MCP-1	[27,28,29,30,31]
Adalimumab	Fully human IgG1 monoclonal antibody specific for human TNF-α	Binds specifically to TNF-α → blocks its interaction with the p55 and p75 cell surface TNFR (TNFR1 and TNFR2). In the presence of complement, lyses cells that express at the surface TNF. No binding/inactivation of TNF-β	2002	AS, CD, JIA, PsO, PsA, RA, UC, Uveitis, nr-axSpA	Subcutaneously every other week	Decreases the expression of adhesion molecules ELAM-1, VCAM-1, ICAM-1, IL-10, IL-12 Decreases the serum levels of MMP-1 and MMP-3 Decreases the serum levels of CRP, ESR	[32,33,34,35,36,37,38,39,40]
Golimumab	Fully human IgG1 κ monoclonal antibody specific for human TNF-α	Selectively neutralizes the biological activity of TNFα by binding to the tmTNF-α + sTNF-α → inhibits the binding of TNFα to TNFR. No binding/inactivation of TNF-β.	2009	AS, PsA, RA, UC, nr-axSpA	Subcutaneously every month	Decreases the serum levels of CRP, IL-6, MMP-3, haptoglobin, ferritin	[41,42,43,44]
Certolizumab pegol	Humanized antibody Fab’ fragment conjugated with two polyethylene glycol molecules, specific for human TNF-α	Selectively neutralizes the biological activity of TNFα by binding to the tmTNF-α + sTNF-α → inhibits the binding of TNFα to TNFR. No binding/inactivation of TNF-β. Does not contain a fragment-crystallizable (Fc) region = does not fix complement or cause antibody-dependent cell-mediated cytotoxicity in vitro.	2008	AS, CD, PsA, RA, nr-axSpA	Subcutaneously; remission induction weeks 0, 2 and 4, maintenance of remission every 4 weeks	Decreases the serum levels of CRP	[45,46,47]

FDA = U.S. Food and Drug Administration; RA = rheumatoid arthritis, JIA = juvenile idiopathic arthritis, PsA = psoriatic arthritis, PsO = plaque psoriasis, AS = ankylosing spondylitis, CD = Crohn’s disease, UC = ulcerative colitis; ICAM-1 = intercellular adhesion molecule-1; VCAM-1 = vascular cell adhesion molecule-1; MCP-1 = monocyte chemotactic protein-1; IL = interleukin; MMP = matrix metalloproteinase; ELAM = endothelial leucocyte adhesion molecule-1 ; VEGF = vascular endothelial growth factor; ESR = erythrocyte sedimentation rate; nr-axSpA = non-radiographic axial spondyloarthritis.

## 2. TNF-α Mechanism of Action

TNF-α is produced in many cells but mostly in activated macrophages, T cells, NK cells, endothelial cells, and fibroblasts [48]. The membrane-bound pro-TNF-α, also called transmembrane TNF-α (tmTNF-α), is proteolyzed by a metalloproteinase, TNF-converting enzyme (TECA), in soluble TNF-α (sTNF-α). Both tmTNF-α and sTNF-α can bind to the TNF receptors type 1 and 2 (TNFR1 and TNFR2) and activate consecutively several signaling pathways. Even if at a low concentration, TNFR1 is found in almost all cell types, while TNFR2 is found manly in immune cells [49]. 

**Figure 1:**
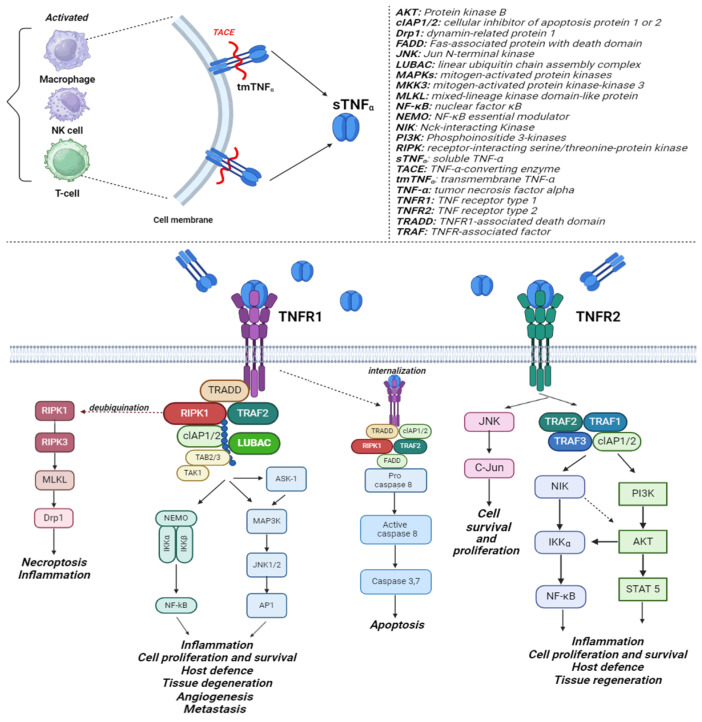
TNF-α mechanism of action. For details, see the text above. Created with biorender.com.

Activation of TNFR1 triggers the interaction with TNFR1-associated death domain (TRADD) and consecutive binding to other proteins, such as TNFR-associated factor 2 (TRAF2), receptor-interacting serine/threonine-protein kinase 1 (RIPK1), transforming growth factor beta-activated kinase 1 (TAK1) and the adapter proteins TAB1 and TAB2, linear ubiquitin chain assembly complex (LUBAC), and cellular inhibitor of apoptosis protein 1 and 2 (cIAP1/2) (Figure 1). Using the ubiquitin ligase E3, C-IAP1/2 ubiquitinate RIPK1 and themselves, enabling the binding of LUBAC, which continues to add linear ubiquitin chains. NEMO will then bind to the linear chains, enabling the recruitment of IKKα/β, which leads to IκB ubiquitination and degradation and consecutive activation of the canonical NF-κB pathway [50]. TAB2/3 (TAK1-binding proteins 2 and 3) transport TAK1 to the complex, thus contributing also to the activation of the MAPK signaling pathway and activator protein-1 (AP-1) [50]. These events will lead to the activation of Nf-κB and to subsequent cell survival, inflammation, defense against pathogens, angiogenesis, methoastasis, and tissue degeneration [51,52]. By removing the polyubiquitin chains, RIPK1 can dissociate from TNFR1 and can bind to RIPK3, leading to the formation of a microfilament-like complex called the necrosome; this aggregate phosphorylates the pseudokinase mixed-lineage kinase domain-like (MLKL) and ultimately induces necroptosis [53]. With time, the activated TNFR1 may be endocytosed; in the cytoplasm, the free receptor can form other complexes with TRADD, Fas-associated protein with death domain (FADD), RIPK1, TRAF2, cIAP1/2, and pro-Caspase-8 [54]. Pro-Caspase-8 is proteolytically cleaved and releases active caspase-8, which in turn activates caspase-3 and -7 and triggers apoptosis. 

The TNFR2 receptor does not present a death domain (DD); however, it has a short amino acid sequence that permits the recruitment of adapter protein TRAF1, TRAF2, TRAF 3, and cIAP1/2 (Figure 1). Compared to TNFR1, where its activation led to the canonical activation of the Nf-κB pathway, TNFR2 activates the alternative NF-κB pathway by recruiting the TRAF1/2/3-cIAP1/2 complexes [54]. Specifically, the induced depletion of these cytosolic complexes results in the accumulation of active NF-κB-inducing kinase (NIK), followed by NIK-mediated phosphorylation of ΙΚΚα and protein p100; the phosphorylated p100 is cleaved into p52, which is then translocated to the nucleus, where it regulates gene transcription [55,56]. Activation of the TNFR2 receptor can also trigger the activation of the PI3K/AKT pathway. As a result, AKT can activate the NF-κB pathway and also increase the phosphorylation of STAT5 [57]. Moreover, recent studies demonstrated that TNFR2 activates the JNK pathway in a TRAF2-independent fashion, leading to the phosphorylation of C-Jun [58]. 

## 3. Research Methods

The data on the neurological adverse effects associated with tumor necrosis factor alpha (TNF-α) inhibitors was obtained after a thorough literature search on PubMed using specific keywords related to the topic: “neurologic”, “TNF-α inhibitors”, “inflammatory”, and “neurodegenerative”. The search included the entire period of TNF-α inhibitor clinical use (1998—present), starting with the approval by the US Food and Drug Administration (FDA) in 1998 of the first marketed compound, infliximab, for the treatment of Crohn’s disease. Only case reports that provided detailed clinical insights and specific instances of neurological adverse effects linked to TNF-α inhibitor therapy were selected. The literature search identified 113 case reports, which were included in the review, statistically assessed, and discussed.

## 4. Results

**Figure 2:**
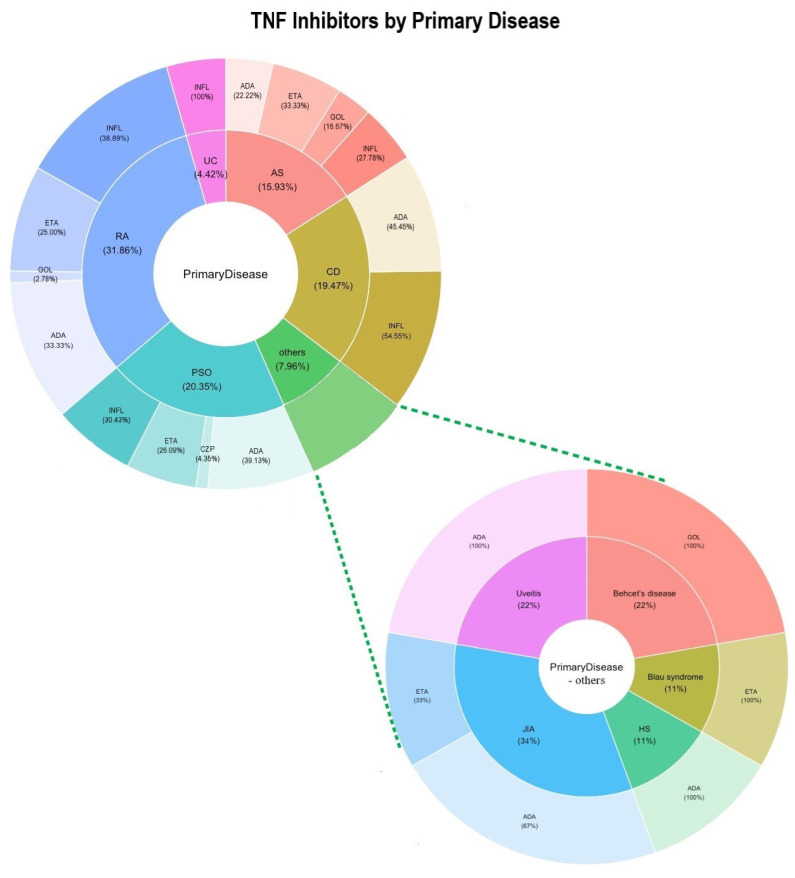
Percentage distribution of TNF-α inhibitors used in various diseases.

Detailed results are presented in Table 2, where data show that 113 cases of neurologic events were identified, with a mean age of 45; 61% of the patients were women and 39% men. The neurological adverse effects occurred either immediately after the first drug infusion or after various periods of time, up to 10 years of drug usage. The most common primary disease involved was rheumatoid arthritis (31.86%), followed by psoriasis (20.35%) and Crohn’s disease (19.47%) (Figure 2); however, a variety of other autoimmune conditions were also the causes for TNF-α inhibitor administration. Infliximab and adalimumab were the most commonly used, each counting for more than 35% of cases; the second most common was etanercept, while the fewest cases were recorded for certolizumab pegol and golimumab, which were introduced later on the pharmaceutical market. No family or personal history of neurologic conditions was reported in 20% of cases, but this result carries poor significance since more than 70% of the cases did not report any investigation in this regard. 

**Figure 3:**
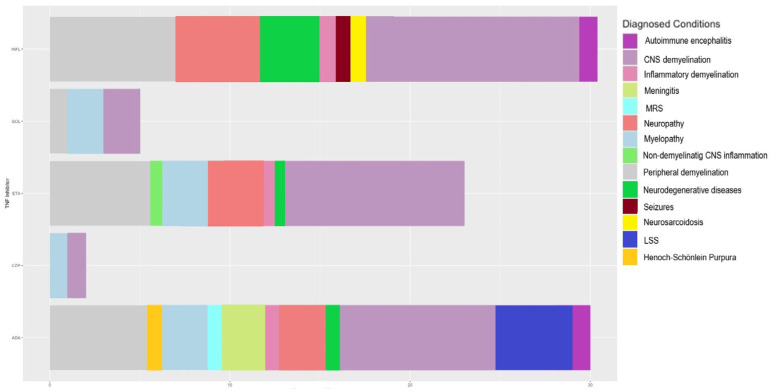
Diagnosed conditions after treatment with TNF-α inhibitors: autoimmune encephalitis (purple), CNS demyelination (light purple), inflammatory demyelination (pink), meningitis (yellow–green), MRS (cyan), multifocal motor neuropathy (red), myelopathy (light blue), non-demyelinating CNS inflammation (light green), peripheral demyelination (grey); neurodegenerative diseases (green); seizures (dark red); neurosarcoidosis (yellow); LSS (blue); Henoch–Schönlein purpura (dark yellow). INFL (infliximab), GOL (golimumab), ETA (etanercept), CZP (certolizumab), ADA (adalimumab).

Neurological events occurred presumably in relation to TNF-α therapy. These events consisted of both demyelinating and non-demyelinating conditions; however, the demyelination reactions prevailed (83% versus 17%). INFL shows a strong association with demyelination, more precisely, with CNS (43%) and peripheral (18%) demyelination; INFL also induced neuropathies (15%), neurodegenerative diseases (7.5%), inflammatory demyelination (6.5%), seizures (5%), and neurosarcoidosis (5%) (Figure 3). ETA displayed a significant portion of CNS demyelination (41%), followed by peripheral demyelination (22%), neuropathies (14.9%), myelopathy (11%), inflammatory demyelination (3.7%), non-demyelinating CNS inflammation (3.7%), and neurodegenerative diseases (3.7%). CZP was mainly associated with myelopathy (50%) and CNS demyelination (50%). GOL induced CND demyelination in 40% of cases and myelopathy (40%), followed by peripheral demyelination (20%). The neurological adverse effects after ADA treatment were mainly CNS (36%) and peripheral demyelination (15%), followed by LSS (10%) neuropathies (7.7%), meningitis (7.7%), myelopathy (7.7%), autoimmune encephalitis (5.1%), Henoch–Schönlein purpura (2.7%), inflammatory demyelination (2.7%), MRS (2.7%), and neurodegenerative diseases (2.7%) (Figure 3). 

**Figure 4:**
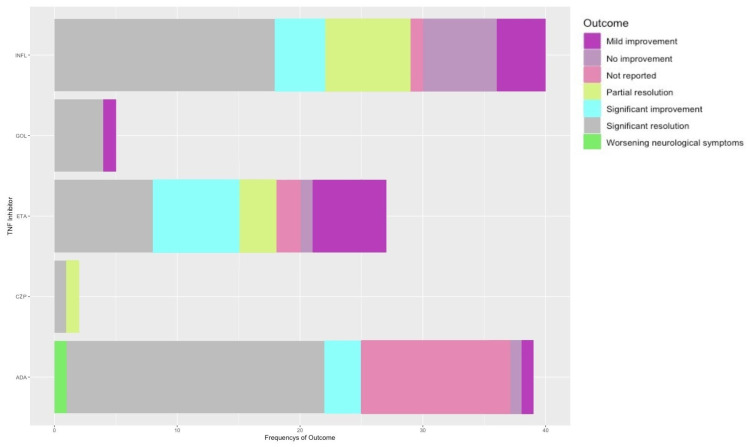
Primary disease outcome after treatment with TNF-α inhibitor: mild improvement (purple), no improvement (light purple), not reported (pink), partial resolution (yellow–green), significant improvement (cyan), significant resolution (gray), worsening neurological symptoms (green). INFL (infliximab), GOL (golimumab), ETA (etanercept), CZP (certolizumab), ADA (adalimumab).

The TNF-α inhibitor discontinuation led, in the majority of cases, to partial or total resolution of the secondary diagnosed condition; only one case was reported where despite the adverse effects, adalimumab was continued due to its high efficiency in treating the primary condition, Crohn’s disease. INFL showed a balanced distribution of outcomes, with significant resolution as the predominant one (53%); the rest were as follows: 10% mild improvement, 15% no improvement, 2.5% not reported, partial resolution 18%, and significant improvement 2.5% (Figure 4). ADA was the only TNF-α inhibitor that led to the worsening of the neurological symptoms (2.6%) in parallel with a high frequency of significant resolution cases (59%). ETA displays a variety of outcomes with numerous instances of significant resolution (34.5%), significant improvement (24.2%), and mild improvement (22%) in the outcomes of the patients. CZP was only associated with partial (50%) and significant resolution (50%). GOL discontinuation resulted in significant (80%) and mild improvement (20%) of secondary disease (Figure 4).

## 5. Discussion

The administration of TNF-α inhibitors can be associated with various types of neurological damage, although the risk of such events is rather low. In numerous cases, TNF-α inhibitors triggered inflammatory reactions in the CNS with associated demyelinating or non-demyelinating conditions. However, there is much debate about whether this treatment is really the cause of such reactions since inflammatory events in the CNS have been reported even before the introduction of biological therapies in autoimmune diseases which by themselves may be responsible for CNS inflammatory processes. It is of particular importance to recognize the intrinsic neurological manifestations of the autoimmune condition itself in order to correctly establish a causality relationship between administered drugs and clinical symptoms; for example, inflammatory bowel diseases are mostly associated with cerebrovascular or demyelinating CNS disease, as well as peripheral neuropathy [150].

One study conducted in 2011 in 10 patients revealed the development of demyelination events as a result of TNF-α inhibitor therapy with a clear distinction from sporadic multiple sclerosis; most symptoms occurred within one year from the beginning of the therapy [151]. In 2020, Kunchock et al. evaluated a lot of 212 patients with autoimmune diseases divided in two groups according to the presence/absence of CNS inflammation; the study reported that the administration of TNF-α inhibitors was clearly associated with higher risk of both demyelinating or non-demyelinating events, in particular in patients diagnosed with rheumatoid arthritis, but could not conclude whether this association occurred separately or represented an exacerbated inflammation process [152]. White matter lesions in the brain parenchyma were identified through magnetic resonance imaging in a patient exhibiting neurological symptoms such as disorientation and confusion which were diagnosed as posterior reversible encephalopathy syndrome that was linked mainly to the existence of an autoimmune condition, rheumatoid arthritis, but also to the administration of immunosuppressive medication, in this case infliximab [153]. The loss of brain parenchyma integrity following TNF-α inhibitor therapy was confirmed through advanced MRI techniques, but neither inflammation nor demyelination could be identified as potential causes [154]; similarly, a fulminant and fatal CNS adverse event occurred after the first dose of infliximab in a pediatric patient who was diagnosed with ischemic and hemorrhagic central processes instead of demyelination or inflammation but was labeled as drug-related [155].

**Figure 5:**
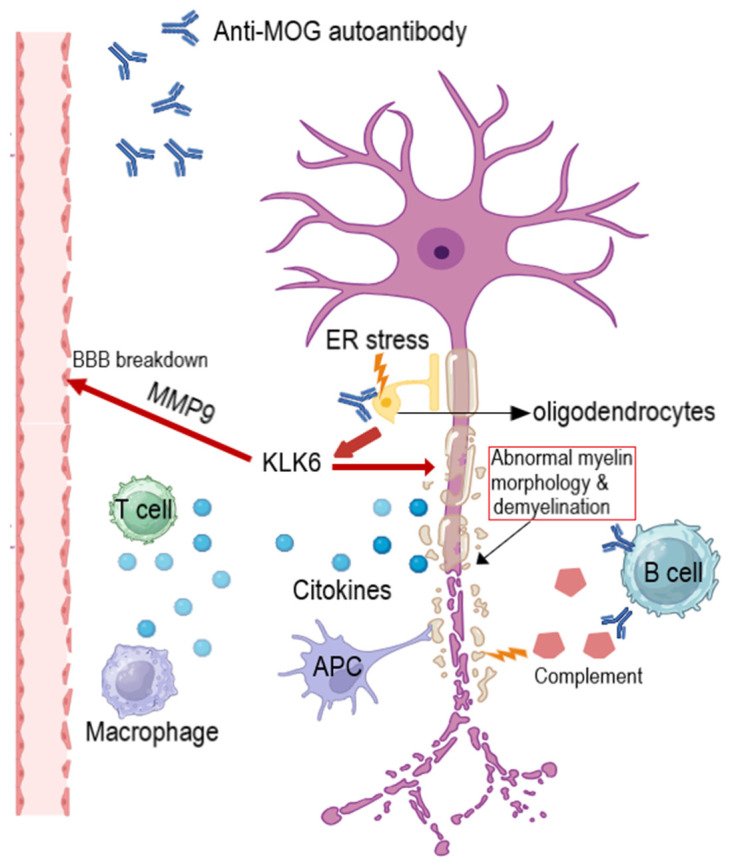
MS pathology. T cells, macrophages, and antigen-presenting cells (APCs) enter the central nervous system (CNS) and secrete cytokines that damage the oligodendrocytes, cells that produce myelin in the CNS. B cells can produce myelin-specific antibodies which will lead to complement cascade activation and membrane–attack complex formation that further damages the oligodendrocytes. A new hypothesis suggests that anti-myelin oligodendrocyte glycoprotein (anti-MOG) autoantibody will induce stress in the oligodendrocyte endoplasmic reticulum (ER). This event will induce morphological abnormalities in oligodendrocytes and demyelination in the broad sense. Moreover, oligodendrocytes secrete Kallikrein 6 (KLK6) and matrix metalloprotease (MMP)-9, which will damage the blood–brain barrier (BBB) and exacerbate the pathology of multiple sclerosis [156]. Created with biorender.com.

In our analysis, most adverse effects were reported for etanercept, infliximab, and adalimumab; their longer usage period in comparison to golimumab and certolizumab may be a contributing factor. Also, most neurological conditions were classified as demyelinating events, including clear diagnosis as multiple sclerosis. MS is a complex pathology that involves demyelination and axonal degeneration at the central level mainly due to immunological factors; the pathogenesis of the disease consists of an autoimmune attack against myelinated axons mediated by inflammatory cytokines and chemokines that facilitate the recruitment of infiltrating inflammatory cells, which are further activated in the CNS, where they react with immune and neuronal cells, thus triggering demyelination and axonal degeneration [156]. The mechanisms identified in the pathogenesis of MS are illustrated in Figure 5.

Demyelinating events may occur as a development of the autoimmune disease itself; in fact, the occurrence of a single acute neurological symptom, which is common in MS, may be an early sign of an autoimmune condition, thus making the differential diagnosis highly challenging [157]. In our analysis, the most frequently encountered primary diseases were rheumatoid arthritis, Crohn’s disease, and psoriasis. In an excellent review published in 2018, Atzeni et al. summarized the direct and indirect effects of rheumatoid arthritis on the CNS [158]. Briefly, the systemic inflammation in RA may directly be responsible for meningitis, vasculitis, and rheumatoid nodules, although such events are rather uncommon; indirectly, the prolonged systemic inflammation in RA may facilitate the development of CNS comorbidities, including vascular, neurodegenerative and demyelinating events. However, demyelinating conditions, including MS, rarely occur in RA patients who have not been treated with TNF-α inhibitors; also, the occurrence of extrapyramidal syndromes is low, but this could be attributed to anti-inflammatory drugs used as chronic treatment in RA. RA may also be accompanied by seizures, which display an inverse relation with the use of NSAIDs; dementia and other neuro-psychiatric pathologies also occur with relatively high frequency in comparison to the general population. Generally, various types of peripheral neuropathies may develop in autoimmune rheumatic diseases; in our analysis, eight cases of Guillain–Barré syndrome were identified while the diagnosis of peripheral neuropathy was established in more than 35% of all cases. In Crohn’s disease, about 3% of the patients experience neurological involvement consisting of peripheral nerve disease, myelopathy, myopathy, and cerebrovascular events; non-enhancing lesions of the white matter of ischemic, atherosclerotic, vasculitic, or demyelinating nature can be seen in asymptomatic IBD patients [159]. CD may co-exist with MS and a family/personal history of one of the two diseases may increase the risk of developing the other; this may be attributed to genes shared by several autoimmune pathologies which subsequently cluster in certain families and to contributing environmental factors. Regarding psoriasis, the pathogenesis of this autoimmune skin pathology shows multiple links with several neurodegenerative conditions, presumably due to the common germ layer, the ectoderm; additionally, the common background includes genetic factors, similar inflammatory mediators, oxidative stress, and various associations with metabolic disorders [160]. In ankylosing spondylitis which also occurs rather frequently as the primary disease in our study, the most common neurological symptoms are caused by spinal cord involvement due to impingement of bones and spinal stenosis [161]; however, subclinical neurological complications are more frequently seen compared to clinically manifested symptoms [162]. 

The common measure in all reviewed cases was the discontinuation of TNF-α inhibitor therapy with or without the administration of additional treatment, usually consisting of corticosteroids, human immunoglobulin, and plasmapheresis. The most prevalent outcome after the discontinuation of the TNF-α inhibitor was the resolution or partial resolution of all neurological symptoms within various periods, usually ranging from a few days to a few months; only 16.3% of all cases showed no/limited response with one case of worsening neurological symptoms. Such symptom progress supports the hypothesis that biological therapy is directly related to the occurrence of neurological events. However, in all three cases of ALS development, the TNF-α inhibitor discontinuation did not induce any symptom improvement, and the disease progress finally led to the patient’s death by respiratory insufficiency. 

**Figure 6:**
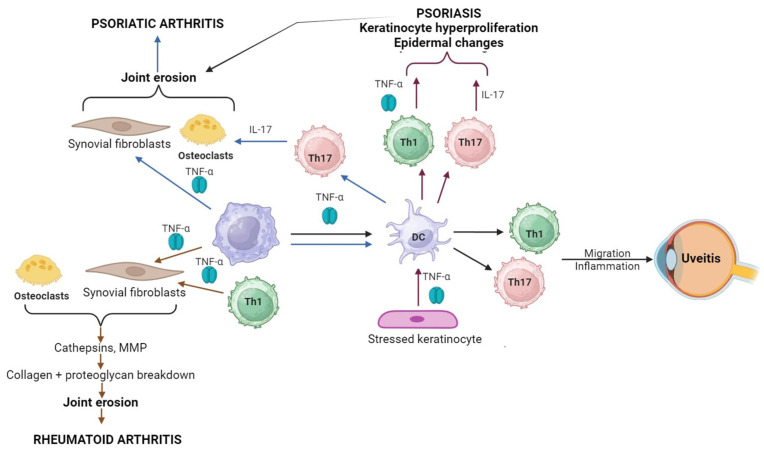
The role of TNF-α in PSO, RA, PsA, and uveitis. In RA, macrophage and Th1 cells secrete TNF-α, activating the synovial fibroblasts, which leads to excessive production of cathepsins and matrix metalloproteinases (MMPs). This overproduction will result in cartilage and bone destruction and consequently joint erosion. In PSO, the stressed keratinocytes secrete TNF-α, which activates dendritic cells (DCs), inducing the activation of Th1 cells and Th17 cells. The activated T cells secrete TNF-α and IL-17, which cause epidermal changes and keratinocyte hyperproliferation. Activation of DCs by TNF-α secreted by macrophages induces migration of T cells and inflammation responsible for uveitis. In PsA, macrophages secrete TNF-α, which activates in parallel the synovial fibroblasts and DCs. DC activation will further activate Th17, which will produce keratinocyte hyperproliferation and epidermal changes and activate osteoclasts. Created with biorender.com.

TNF-α is a cytokine with pleiotropic effects on various cells thus exerting a crucial role in triggering a normal immune response; its regulating activity makes it a key player in the pathogenesis of several inflammatory and autoimmune pathologies. It is a homotrimer protein containing 157 amino acids and generated by activated immune cells; in turn, TNF-α induces the production of other inflammatory mediators. TNF-α is synthesized as the transmembrane form which is processed into its soluble form in order to be released. Two types of receptors have been identified, TNFR1 and TNFR2; the first is present in all cells and tissues, being the key signaling receptor, while the second occurs in immune cells and performs limited functions. Although it binds to both types, the transmembrane TNF-α acts mainly through TNFR2, while its soluble form activates both types of receptors [163]. Its involvement in the pathogenesis of various autoimmune diseases encompasses multiple mechanisms, as depicted in Figure 6.

TNF-α inhibitors have been proposed as a treatment in MS based on the key role played by the TNF-α cytokine in MS pathogenesis; however, multiple studies have shown that not only did TNFα inhibitors fail to stop disease progress, but they even caused symptom exacerbation [164]. Recent studies, however, highlighted the difference between the two types of TNF receptors in terms of biological effects; while TNFR1 exerts pro-inflammatory and apoptotic effects, TNFR2 is associated with immunoregulatory, regenerative, and neuroprotective activities [165]. Therefore, a potential explanation for treatment failure in MS, as well as the adverse neurological events that occurred in other therapies, is the non-selective effect of TNF-α inhibitors currently in use, which counteracts the anti-inflammatory activity mediated by TNFR2 receptors. As support for this hypothesis stand the therapeutic effects exerted by atrosimab, a selective TNFR1 inhibitor, in neurodegenerative diseases in animal models [165]. Similarly, the preclinical evaluation of other TNFR1 inhibitors or DN-TNF muteins revealed superior outcomes to global TNF-α blockers in terms of less severe side effects or the potential to treat pathologies such as MS or other neurodegenerative conditions where non-selective TNFα inhibition is not recommended [166].

Other hypotheses regarding the controversial behaviors of TNF-α inhibitors were summarized in an excellent review conducted by Kaltsonoudis et al.; briefly, the following theories have been formulated: (1) TNF-α inhibitors increase the T-cell response by multiple mechanisms, followed by their entrance into the CNS and subsequent demyelination; (2) TNF-α inhibitors alter the downstream cytokine response by decreasing IL-10 and increasing IL-12 production, thus facilitating demyelination; (3) TNF-α inhibitors cause a peripheral reduction in TNF-α, while brain levels remain relatively unchanged due to the blood–brain barrier, which is difficult to cross; this may increase the level of TNFα in the brain and/or TNFR expression, thus inducing demyelinating lesions, and (4) TNF-α inhibitors may activate latent infections able to trigger autoimmune demyelinating events [167].

Controversially, we were able to identify a case where etanercept was successfully used for the treatment of ankylosing spondylitis combined with demyelinating myelitis; however, considering the already known risks of biological therapy, the administered dose was half (25 mg/week) of the routine dose (50 mg/week). The follow-up MRI scan (after 35 days) revealed a reduction in the vertebrae demyelinating lesion without the occurrence of new ones [168]; a further reduction in the dose to 25 mg every two weeks did not induce a relapse of either pathology. Another controversial case was reported, describing a patient who developed the Guillain–Barré syndrome after the initiation of infliximab therapy for psoriasis and ankylosing spondylitis [169]; the infliximab therapy was discontinued but then reinstated due to a severe relapse, however, at a lower dose that managed to control the autoimmune conditions. These reports suggest that the occurrence/severity of neurological events during TNF-α inhibitor therapy may be also related to the administered dose which was sufficient to control patient’s condition but low enough to avoid neurologic disease.

## 6. Conclusions and Future Perspectives

This study summarizes the neurological events reported during and after the administration of TNF-α inhibitors as treatment for various primary diseases. The profound involvement of TNF-α in the pathogenesis of MS suggested the potential of using TNF-α inhibitors as treatment for neurodegenerative diseases; far from producing beneficial effects, the drugs even exacerbated the existing symptoms. The debate regarding the true link between the occurrence of neurological events and the use of TNF-α inhibitors started almost immediately after the approval of the first such drug, etanercept, more than 20 years ago. During this time, both demyelinating and non-demyelinating events have been reported, with a large plethora of symptoms for all five approved TNF-α inhibitors. A number of theories were suggested in the attempt to explain the underlying mechanisms of such events; the most recent one is based on the existence of the two types of TNF-α receptors with opposite biological effects that triggered the discovery of selective TNFR1 inhibitors which induced superior outcomes to global TNF-α antagonists. The reported beneficial effects of TNF-α inhibitors in demyelinating diseases, although rare compared to the vast majority of neurological adverse effects, seem to be related to the administered dose, the authors concluding that it was high enough to control the primary disease and low enough to avoid triggering neurological events. Regardless, the general opinion in the field is that TNF-α inhibitors should be avoided in patients with a personal or familial history of demyelination (such as patients having first-degree relatives diagnosed with MS) due to their genetic proneness to develop such conditions even in the absence of an anti-TNF treatment; it is still possible that some patients have developed the disease anyway and the administration of the inhibitors only hastened it. Additionally, in countries where only TNF-α inhibitors are approved as advanced treatments in rheumatic diseases, clinicians should be aware of the potential neurological side effects and even the unmasking of a latent neurodegenerative condition with severe consequences; furthermore, a family history of autoimmune conditions may indicate increased susceptibility towards CNS demyelination, aspects that must be considered during the initial clinical evaluation of the patient. Constant monitoring is also necessary for patients undergoing TNF-α inhibitor therapy, in particular for those suffering from illnesses found to be correlated with more frequent side effects; such monitoring includes laboratory tests where dermatology patients exhibit less frequent abnormal results than gastroenterology or rheumatology patients, therefore requiring less frequent testing. Usually, laboratory evaluation should be performed at baseline and after 3, 6, and 12 months of treatment, followed by annual evaluation or more frequently depending on comorbidities, other medications, or medical experience (10.5144/0256-4947.2022.309). Full physical examination, brain MRI, and neurophysiology tests are essential both for the initial patient evaluation in order to identify preexisting demyelinating diseases and for the follow-up and monitoring of potential developing neurological complications. If any neurological symptoms occur, the patient should undergo a thorough neurological examination, including MRI scanning, that can confirm or exclude demyelination; if demyelination is confirmed, the TNF-α inhibitor should be discontinued and therapy adjusted to patient needs. Future studies will presumably lead to the discovery of other TNFR1 selective inhibitors with improved therapeutic outcomes, therefore reducing the risk of neurological adverse effects.

## Figures and Tables

**Table 2:** Details on the neurologic events after treatment with TNF-α inhibitors.

	Primary Disease	TNF-α Inhibitor	Age/Sex	Family History of Neurological Diseases	Neurological Symptoms/Diagnosed Conditions	Therapeutic Measures	Outcome	Ref.
1.	RA	INFL	Not reported/M	Not reported	ALS	Discontinuation Riluzole	Progressive ALS	[59]
2.	AS	INFL	55/M	Not reported	ALS	Discontinuation Riluzole, alphatocopherol	Progressive ALS; death by respiratory insufficiency	[60]
3.	RA	INFL	65/F	Not reported	ALS	Discontinuation Riluzole not tolerated	Progressive ALS; death by respiratory insufficiency	[61]
4.	CD	ADA	53/F	Not reported	Demyelinating lesions/autoimmune encephalitis	Discontinuation Intravenous steroids, immunoglobulin	Resolution	[62]
5.	AS	ADA	30/F	Not reported	Demyelinating lesions/autoimmune encephalitis (Bickerstaff brainstem encephalitis)	Discontinuation Methylprednisolone, intravenous gamma globulin (IVIg), rituximab	Partial resolution	[63]
6.	CD	ADA	20/F	Not reported	Peripheral neuropathy/Henoch–Schönlein purpura with neurological involvement	Discontinuation ADA replaced with ustekinumab/methylprednisolone	Resolution	[64]
7.	CD	INFL	64/F	Neurofibromatosis	Acute neuropathy, encephalopathy	Discontinuation Polyvalent immunoglobulin	Partial resolution	[65]
8.	CD	INFL	27/F	Not reported	Non-demyelinating inflammatory CNS events/anti-NMDAR encephalitis	INFL replaced with ustekinumab Rituximab	Resolution	[66]
9.	RA	INFL	72/M	Not reported	Demyelinating progressive multifocal leukoencephalopathy	Discontinuation	Persistent neurologic and cognitive deficits	[67]
10.	CD	INFL	45/F	Not reported	Acute disseminated encephalomyelitis	Discontinuation Methylprednisolone	Resolution	[68]
11.	RA	ADA	66/F	Not reported	Demyelinating progressive multifocal leukoencephalopathy	Discontinuation	Worsening neurological symptoms	[67]
12.	RA	ADA	66/M	Not reported	Demyelinating leukoencephalopathy	Discontinuation Methylprednisolone Meloxicam	Partial resolution	[69]
13.	AS	GOL	45/F	Not reported	Demyelinating posterior reversible encephalopathy syndrome	Discontinuation, anti-convulsant therapy (levetiracetam)	Resolution	[70]
14.	RA	ETA	74/F	Not reported	Demyelinating progressive multifocal leukoencephalopathy	Discontinuation Supportive therapy	Slight improvement	[71]
15.	RA	ETA	47/F	Not reported	Leptomeningitis	Discontinuation Methylprednisolone, azathioprine, levetiracetam, valproic acid.	Significant resolution	[72]
16.	RA	ADA	77/M	Not reported	Hypertrophic pachymeningitis	Discontinuation, Prednisone	Resolution	[73]
17.	JIA	ADA	18/F	Not reported	Aseptic meningitis	Discontinuation Methylprednisolone	Resolution	[74]
18.	RA	ADA	52/F	Not reported	Acute myelopathy/paraplegia	Discontinuation Methylprednisolone (oral/i.v)	Partial resolution	[75]
19.	CD	ADA	52/F		Chronic myelitis		Partial resolution	
20.	RA	ADA	42/M		Chronic myelitis		No response	
21.	RA Lupus	CZP	54/F		Chronic myelitis		Partial resolution	
22.	Blau syndrome	ETA	13/M	Not reported	Transverse myelitis	Discontinuation Methylprednisolone, prednisone	Resolution	[76]
23.	Behcet’s disease	GOL	34/M	Not reported	Demyelinating lesions	Discontinuation Steroid therapy	Resolution	[77]
24.	Behcet’s disease	GOL	51/F	Not reported	Multifocal myelitis	Discontinuation Steroid therapy	Resolution	[78]
25.	CD	INFL	40/M	Not reported	Extensive longitudinal myelopathy	Discontinuation Methylprednisolone, followed by oral prednisolone	Slight improvement	[79]
26.	AS	ADA ETA	50/M	Not reported	Rapidly progressive dementia	Discontinuation	Resolution	[80]
27.	RA	ADA	40/F	None	CNS demyelination	Discontinuation Methylprednisolone	Resolution	[81]
28.	PSO	ADA	36/F	None	CNS demyelination	Discontinuation	Resolution	[82]
29.	PSO	ADA	37/F	None	CNS demyelination	Discontinuation Intravenous immunoglobulin, oral corticosteroids	Resolution	[83]
30.	RA	ADA	39/F	Not reported	Chronic demyelinating neuropathy	Discontinuation	Resolution	[84]
31.	CD	ADA	34/F	None	Inflammatory demyelination	Discontinuation Steroid therapy	Slight improvement	[85]
32.	HS	ADA	29/M	Not reported	Demyelinating radiologically isolated syndrome	Discontinuation	Resolution	[86]
33.	JIA	ADA	14/M	Not reported	CNS active inflammatory process (no demyelination lesions)	Discontinuation High-dose corticosteroids	Partial resolution	[87]
34.	PSO	ADA	51/F	None	MS	Discontinuation high-dose corticosteroids	Partial resolution	[88]
35.	AS	ETA	34/M	None	CNS demyelination	Discontinuation	Slight improvement	[89]
36.	AS	ETA	26/F	Not reported	MOGAD	Discontinuation Methylprednisolone	Significant improvement	[90]
37.	AS	ETA	44/F	Not reported	Tumefactive CNS inflammatory demyelination	Discontinuation High-dose steroids	Significant improvement	[91]
38.	AS	ETA	36/M	MS (brother)	Acute transverse myelitis	Discontinuation Methylprednisolone	Resolution	[92]
39.	Juvenile RA	ETA	21/F	None	MS Optic neuritis	Discontinuation Methylprednisolone	Partial resolution	[93]
40.	PSO	ETA	55/M	None	MS	Discontinuation Methylprednisolone	No improvement	[94]
41.	AS	ADA	44/M	None	Demyelinating brain lesions	Discontinuation	Not reported	
42.	RA	ETA	26/F	Not reported	MS	Not reported	Not reported	[95]
43.	AS	GOL	41/M	None	Fulminant CNS demyelination	Discontinuation Steroid therapy Plasmapheresis	Resolution	[96]
44.	PSO	INFL	47/F	MS (sister)	MS	Discontinuation Steroid therapy	Partial resolution	[97]
45.	PSO	INFL	27/F	None	CNS demyelination	Discontinuation Steroid therapy Rituximab	Partial resolution	[98]
46.	CD	INFL	46/M	None	Inflammatory demyelinating polyneuropachronicth	Discontinuation Intravenous human immunoglobulin	Resolution	[99]
47.	CD AS	INFL	18/F	Not reported	CNS demyelination	Discontinuation	No improvement	[100]
48.	AS Uveitis	INFL	38/M	Not reported	Demyelinating brain lesions	Discontinuation	Resolution	[101]
49.	RA	INFL	56/M	Not reported	CNS demyelination	Discontinuation High-dose dexamethasone	Resolution	[102]
50.			66/F	Not reported		Discontinuation Oral prednisone	Significant improvement	
51.	CD	INFL	19/F	None	CNS demyelination	Discontinuation	Resolution	[103]
52.	Uveitis JIA	ADA	21/F	Not reported	MS	Discontinuation Cladribine	Resolution	[38]
53.	PSO	ADA	56/F	Not reported	MS aseptic meningitis	Discontinuation	Resolution	[104]
54.	RA	ADA	68/F	None	MS	Discontinuation	Resolution	[105]
55.	Autoimmune Uveitis	ADA	23/M	MS (family)	MS	Discontinuation Methylprednisolone, oral prednisolone	Resolution	[39]
56.	AS	ADA	36/M	None	MS	Discontinuation Methylprednisolone, oral prednisolone	Resolution	[106]
57.	CD	INFL	47/F	None	MS	Discontinuation	Resolution	[107]
58.	UC	INFL	35/F	MS (family)	MS	Discontinuation	Not reported	[108]
59.	AS	INFL	48/M	Not reported	MS	Discontinuation	Resolution	[109]
60.	PSO	ADA	41/M		CNS demyelination	Discontinuation	Resolution	
61.	PSO	ETA	38/F		MS	Discontinuation Interferon beta-1a	Not reported	
62.	RA	ADA	64/F		MS	Discontinuation	Not reported	
63.	AS	INFL	34/F		Clinical isolated syndrome	Discontinuation	Resolution	
64.	PSO	INFL	57/M		Peripheral demyelinating neuropathy	Discontinuation	Resolution	
65.	PSO	ETA	48/M	Not reported	MS	Discontinuation	Resolution	[110]
66.	PSO	INFL	62/F	Not reported	Optic neuritis	Discontinuation Methylprednisolone	No improvement	[111]
67.	PSO	INFL	56/F	Not reported	CNS demyelination	Discontinuation Methylprednisolone	Significant improvement	[112]
68.	PSO	CZP	44/M	Not reported	Cranial nerve III demyelination	Discontinuation methylprednisolone	Resolution	[113]
69.	RA	ETA	64/F	Not reported	Myelitis optic neuritis	Discontinuation Methylprednisolone	Resolution	[114]
70.	CD	ADA	27/F	Not reported	Internuclear ophthalmoplegia	Discontinuation Steroid therapy	Resolution	[115]
71.	RA	INFL	53/F	Not reported	Optic neuritis	Discontinuation Steroid therapy	Resolution	[116]
72.	RA	ETA	40/F	Not reported	Neurosarcoidosis	Discontinuation Methylprednisolone, oral prednisolone	Partial resolution	[117]
73.	RA Uveitis	ETA	33/F	Not reported	Neurosarcoidosis	ETA replaced with INFL Steroid therapy	Significant improvement	[118]
74.	RA	INFL	50/F	None	Polineuropathy	Discontinuation Intravenous gammaglobulin	Slight improvement	[119]
75.			85/F	Mild sensory loss		Discontinuation Steroid therapy	Partial resolution	
76.			68/F	Not reported		Discontinuation	Slight improvement	
77.	PSO	ADA	65/F	Not reported	Acute bilateral symmetric phrenic neuropathy	Discontinuation Oxygen	Resolution	[120]
78.	RA	ETA	45/F	Not reported	Chronic inflammatory demyelinating polyneuropathy	Discontinuation Intravenous gammaglobulin	Partial resolution	[121]
79.		INFL	49/M	Not reported				
80.	PSO	ADA	53/F	GBS	Chronic inflammatory demyelinating polyradiculoneuropathy	Discontinuation Intravenous immunoglobulin	Resolution	[122]
81.	RA	ADA	52/M	Idiopathic Parkinson’s disease	DADS neuropathy	Discontinuation	Resolution	[123]
82.	AS	GOL	48/M	ALS (family)	Bilateral retrobulbar optic neuropathy	Discontinuation Methylprednisolone, oral prednisolone	No improvement	[124]
83.	PSO	ADA	42/M	Not reported	Chronic inflammatory demyelinating polyneuropathy	Discontinuation intravenous immunoglobulin	Resolution	[125]
84.	PSO	INFL	64/M	Not reported	Sensory polyradiculopathy	Discontinuation Intravenous immunoglobulin	Resolution	[126]
85.	RA	INFL	32/F	Not reported	Small-fiber neuropathies	Discontinuation	Partial resolution	[127]
86.			73/F	Not reported		Discontinuation Symptomatic treatment	Partial resolution	
87.			55/F	Not reported		Discontinuation	Significant improvement	
88.	AS	INFL	54/M	Not reported	MMNCB	Discontinuation	Significant improvement	[128]
89.	UC	INFL	30/M	None	Multifocal motor neuropathy	Discontinuation Intravenous immunoglobulin	Resolution	[128]
90.	JIA	ETA	12/F	Not reported	Chronic inflammatory demyelinating neuropathy	Discontinuation Intravenous immunoglobulins, steroids, plasma exchange	Limited response	[129]
91.	RA	ADA	44/F	Not reported	Inflammatory demyelinating neuropathy	Discontinuation Intravenous immunoglobulin	Partial resolution	[130]
92.	CD	ADA	37/M	Not reported	Chronic inflammatory demyelinating neuropathy	Discontinuation Intravenous immunoglobulins, steroids	Significant improvement	[131]
93.	CD	INFL	60/F	None	Seizures	Discontinuation	Resolution	[132]
94.	CD	INFL	74/M	Not reported	Seizures	Discontinuation	Resolution	[133]
95.	RA	ETA	74/F	Not reported	CJD	Discontinuation	Resolution	[134]
96.	PSO	INFL	40/M	Not reported	LSS	Discontinuation Intravenous immunoglobulin	Partial resolution	[135]
97.	UC	INFL	44/M	None	LSS	Discontinuation Intravenous immunoglobulin	Partial resolution	[136]
98.	CD	INFL	24/F	Not reported	LSS	Discontinuation Intravenous immunoglobulin	Resolution	[137]
99.			35/F	Not reported				
100.	RA	INFL	77/F	Not reported	MFS	Discontinuation High-dose steroids, gabapentine	Slow improvement	[138]
101.	UC	INFL	43/F	Not reported	MFS	Discontinuation	Resolution	[139]
102.	PSO	ETA	42/F	Not reported	MFS	Discontinuation	Slow improvement	[140]
103.	CD	ADA	64/M	Not reported	GBS	Discontinuation	Resolution	[141]
104.	PSO	ADA	45/M	Not reported	GBS	Discontinuation	Partial resolution	[142]
105.	RA	ADA	50/F	Not reported	GBS	Discontinuation Prednisone, gabapentin	Partial resolution	[143]
106.	CD	ADA	37/M	Not reported	GBS	Discontinuation Intravenous immunoglobulin	Partial resolution	[144]
107.	UC AS	INFL	47/F	Not reported	GBS	Discontinuation intravenous immunoglobulin	Resolution	[145]
108.	CD	ADA	71/M	Not reported	GBS	Discontinuation Intravenous immunoglobulin, Methylprednisolone, plasmapheresis	Partial resolution	[146]
109.	CD	ADA	33/F	Not reported	GBS	Continuation of ADA treatment Intravenous immunoglobulin	Significant improvement	[147]
110.	AS	ETA	50/M	Not reported	GBS	Discontinuation Intravenous immunoglobulin, plasmapheresis	Resolution	[148]
111.	PSO	ETA	53/M	None	MS	Discontinuation Interferonβ Baclofen	Significant improvement	[149]
112.	PSO		42/M	None	MS	Discontinuation	Significant improvement	
113.	AS		51/F	Not reported		Discontinuation	Mild improvement	

INFL = infliximab, GOL = golimumab, ETA = etanercept, CZP = certolizumab, ADA = adalimumab, RA = rheumatoid arthritis, JIA = juvenile idiopathic arthritis, HS = hidradenitis suppurativa, AS = ankylosing spondylitis, CD = Crohn’s disease, UC = ulcerative colitis; MS = multiple sclerosis, GBS = Guillain–Barré syndrome, MFS = Miller–Fisher syndrome, LSS = lumbar spinal stenosis, CJD = Creutzfeldt–Jakob disease, MMNCB = multifocal motor neuropathy with conduction blocks, DADS = distal acquired demyelinating symmetric neuropathy, MOGAD = myelin oligodendrocyte glycoprotein antibody disease, ALS = amyotrophic lateral sclerosis. Discontinuation = discontinuation of the TNF-α inhibitor therapy.

## Data Availability

The original contributions presented in the study are included in the article, further inquiries can be directed to the corresponding author(s).
